# Temperature-dependent association between ambient ozone exposure and allergic rhinitis-related outpatient visits: a time-stratified case-crossover study in Henan Province, China

**DOI:** 10.3389/fpubh.2026.1952329

**Published:** 2026-10-20

**Authors:** Dong Ding, Wei Lu, Huina Wang, Leiyu Wang, Jinghong Gao, Tengda Wei, Haohao Liu

**Affiliations:** 1 College of Public Health, Zhengzhou University, Zhengzhou, Henan, China; 2 Department of Public Health, The First Affiliated Hospital of Zhengzhou University, Zhengzhou, Henan, China; 3 School of Public Health, Cheeloo College of Medicine, Shandong University, Jinan, Shandong, China; 4 College of Arts and Sciences, Boston University, Boston, MA, United States

**Keywords:** allergic rhinitis, ambient temperature, case-crossover study, effect modification, outpatient visits, ozone

## Abstract

**Background:**

Ambient ozone (O_3_) may adversely affect the upper airway, but whether temperature modifies its short-term association with outpatient visits for allergic rhinitis remains unclear.

**Methods:**

We conducted a time-stratified case-crossover study of 43,418 outpatient visits for allergic rhinitis at a tertiary hospital in Henan, China, during April–October of 2013–2023. Daily O_3_, PM_2.5_, temperature, and relative humidity (RH) were assigned by residential address using gridded datasets and averaged over lag 0–1 day. Conditional logistic regression estimated odds ratios (ORs) and 95% confidence intervals (CIs) per 10 μg/m^3^ increase in O_3_ across the observed exposure distribution, adjusting for temperature, RH, and PM_2.5_. Temperature modification was assessed using an interaction term between O_3_ and a natural cubic spline function of temperature.

**Results:**

Each 10 μg/m^3^ increase in O_3_ was associated with a 1.2% increase in the odds of an outpatient visit for allergic rhinitis (OR = 1.012, 95% CI: 1.006–1.017). The largest age-specific estimate was observed among patients aged <18 years (OR = 1.030, 95% CI: 1.017–1.044). Temperature significantly modified the association (χ^2^ = 31.2, *P* < 0.001), the OR for O_3_ increased from 1.000 at the 25th percentile of temperature to 1.021 at the 75th percentile.

**Conclusion:**

Short-term O_3_ exposure was associated with higher odds of outpatient visits for allergic rhinitis, with larger estimates at higher temperatures and among patients aged <18 years.

## 1 Introduction

Allergic rhinitis is a common inflammatory disease of the upper airway that can impair sleep, productivity, and quality of life and often coexists with asthma (1). Growing evidence indicates that environmental factors, including ambient air pollution, meteorological conditions, and aeroallergens, may contribute to the onset and exacerbation of allergic rhinitis symptoms and increase related healthcare utilization (2–4).

Ozone (O_3_) is a secondary air pollutant formed through photochemical reactions involving nitrogen oxides and volatile organic compounds in the presence of sunlight. Ambient O_3_ concentrations are influenced by temperature and solar radiation, and O_3_ has become an important warm-season air-quality concern in China (5, 6). As a highly reactive oxidant gas, O_3_ may induce oxidative stress, epithelial barrier damage, and airway inflammation, thereby potentially exacerbating allergic rhinitis symptoms and increasing related healthcare visits (7, 8).

Ambient temperature may influence O_3_-related upper-airway effects through both environmental and biological pathways. Higher temperatures and stronger solar radiation favor photochemical O_3_ formation, increasing the likelihood of concurrent heat and O_3_ exposure. Beyond affecting ambient O_3_ concentrations, warmer conditions may alter outdoor activity patterns and increase breathing rate, potentially affecting the inhaled dose of O_3_. Heat may also promote airway surface dehydration and nasal mucosal irritation, thereby modifying susceptibility to a given level of O_3_ exposure. Epidemiological studies have suggested potential joint or interactive effects of temperature and O_3_ on respiratory outcomes, but evidence specific to allergic rhinitis remains limited (9, 10).

Susceptibility to O_3_-related health effects may vary across population subgroups because of differences in physiology, exposure behavior, and healthcare-seeking patterns. Children and adolescents may be particularly vulnerable because their respiratory and immune systems are still developing, they inhale more air relative to body weight, and they often spend more time outdoors (11). Previous studies have also linked O_3_ exposure to allergic rhinitis and other respiratory outcomes in children and adolescents (12, 13).

Henan Province has a large population and warm-season conditions conducive to O_3_ formation, providing a relevant setting for investigating O_3_-related upper-airway effects. However, evidence on the short-term association between O_3_ exposure and outpatient visits for allergic rhinitis remains limited, particularly regarding temperature-dependent modification and differences across population subgroups. We therefore conducted a time-stratified case-crossover study using outpatient records from a tertiary hospital in Henan during April–October of each year from 2013 to 2023. We aimed to estimate the short-term association between O_3_ exposure and outpatient visits for allergic rhinitis, assess modification by ambient temperature, characterize associations across combinations of O_3_ and temperature, and examine sex- and age-specific estimates.

## 2 Materials and methods

### 2.1 Data sources

This study included outpatient visits for allergic rhinitis recorded at the First Affiliated Hospital of Zhengzhou University, a tertiary hospital in Henan, China, from 2013 to 2023. The electronic outpatient records contained anonymized patient identifiers, visit dates, sex, age, primary diagnoses, and residential addresses. Outpatient visits for allergic rhinitis were identified using the physician-recorded primary diagnosis coded according to the International Classification of Diseases, 10th Revision (ICD-10), in the hospital information system. To avoid counting multiple visits by the same patient within a calendar month, only the first visit per patient per month was retained (Supplementary Figure S1). Because O_3_ pollution in Henan is most prominent during the warm season, the analysis was restricted to visits occurring from April to October of each year.

Ambient air pollution data were obtained from the ChinaHighAirPollutants (CHAP) dataset, which provides spatially continuous gridded estimates across China at a resolution of 0.1° × 0.1°. Daily maximum 8-h average O_3_ was used as the primary exposure, and daily mean PM_2.5_ was included as a co-pollutant (14). Meteorological data were obtained from the ERA5-Land reanalysis dataset provided by the European Center for Medium-Range Weather Forecasts at the same spatial resolution (15). Daily 2-m air temperature and 2-m dew-point temperature data were extracted, and relative humidity (RH) was calculated based on air temperature and dew-point temperature.

Environmental exposures were assigned based on the level of detail available in patients' residential addresses. Detailed residential addresses were geocoded and matched to the nearest 0.1° × 0.1° grid cell, whereas records without detailed addresses were assigned the daily county- or district-level average exposure. The 0.1° × 0.1° grid corresponds to approximately 9–11 km in Henan Province, and multiple individuals could be assigned to the same grid cell. Daily O_3_, PM_2.5_, temperature, and RH were assigned for the case day and matched control days. In the main analysis, 2-day moving averages of all environmental variables were calculated over the current and previous days (lag 0–1) to account for potential short-term cumulative effects.

### 2.2 Study design

A time-stratified case-crossover design was used to evaluate the short-term association between O_3_ exposure and outpatient visits for allergic rhinitis (16, 17). In this design, each patient served as their own control, thereby controlling for time-invariant individual characteristics.

For each outpatient visit, the visit date was defined as the case day. Control days were selected from the same year and calendar month as the case day and were matched by day of the week. For example, if an outpatient visit occurred on May 15, 2021, the control days were the other days falling on the same weekday in May 2021. This time-stratified referent-selection strategy controls for long-term trends, seasonality, and day-of-week effects. A schematic illustration of the time-stratified case-crossover design is shown in Figure 1.

**Figure 1:**
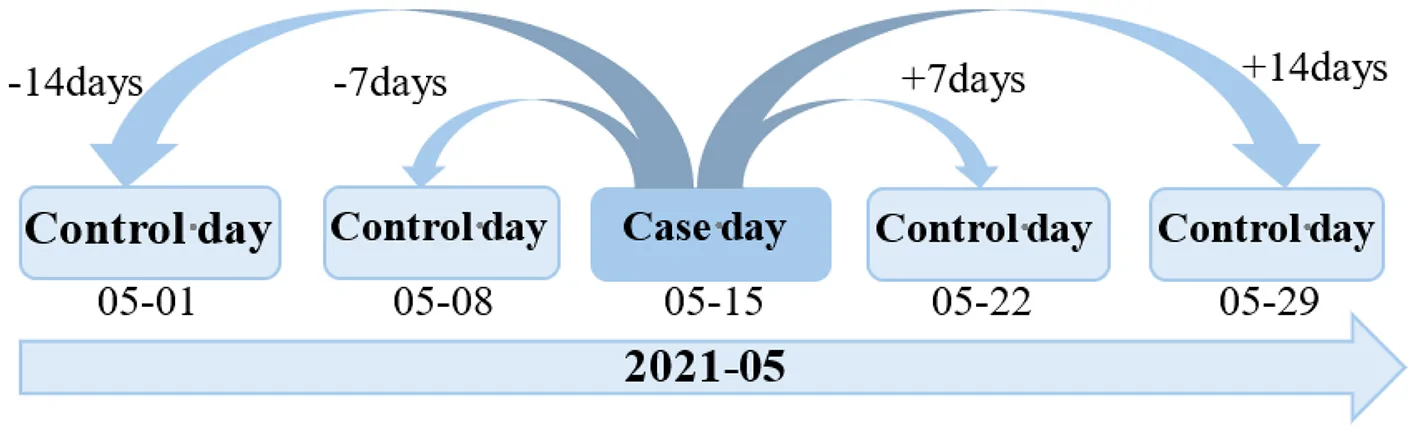
Schematic illustration of the time-stratified case-crossover design.

### 2.3 Statistical analysis

Conditional logistic regression was used to estimate the association between short-term O_3_ exposure and outpatient visits for allergic rhinitis. Each case day and its matched control days constituted a separate stratum. O_3_ was modeled as a linear term, and associations were expressed as odds ratios (ORs) and 95% confidence intervals (CIs) per 10 μg/m^3^ increase in O_3_. The main model included natural cubic spline functions of temperature and RH, each with 3 degrees of freedom (df), and a linear term for PM_2.5_. To explore potential delayed associations, we additionally conducted lag analyses using both single-day lags from lag 0 to lag 14 and moving-average lag windows from lag 0–1 to lag 0–14. The lag 0–1 moving average of O_3_ was used as the main exposure metric. Specifically, lag 0–1 O_3_ was calculated as the average of the daily maximum 8-h average O_3_ on the visit or control day and the previous day: (O_3_, *t* + O_3_, *t*−1)/2.

Although O_3_ was modeled as a linear term in the main analysis for interpretability, we additionally examined the potential non-linear exposure–response association using a natural cubic spline function of lag 0–1 O_3_ with 3 df. O_3_-specific ORs were estimated using the O_3_ concentration associated with the minimum predicted odds as the reference.

The temperature–response association was examined using a natural cubic spline with 3 df, with adjustment for RH, O_3_, and PM_2.5_. Temperature-specific ORs were calculated using the minimum-risk temperature (MRT), defined as the temperature associated with the minimum predicted odds, as the reference. The MRT was estimated from the full-sample temperature–response model and used as the common reference for the overall and subgroup-specific curves.

Effect modification by temperature was assessed by adding an interaction between the linear O_3_ term and the temperature spline to the main model. Models with and without the interaction were compared using a likelihood ratio test. O_3_-related ORs per 10 μg/m^3^ increase were estimated across temperature levels and at the 25th, 50th, and 75th percentiles of temperature.

To characterize associations across combinations of O_3_ and temperature, model-predicted ORs were estimated with O_3_ fixed at its 25th, 50th, and 75th percentiles. All estimates were calculated relative to a common reference combination of the MRT and the 25th percentile of O_3_, allowing direct comparisons across exposure combinations.

Subgroup analyses were conducted by sex and age (<18, 18–64, and ≥65 years), using the same exposure definitions and covariate adjustments as in the main analysis.

### 2.4 Sensitivity analyses

Several sensitivity analyses were conducted to assess the robustness of the findings. For the O_3_ association, the df for the temperature and RH splines were increased from 3 to 4, and the model was refitted without adjustment for PM_2.5_. We also repeated the analysis after restricting the study period to May–September, when warm-season O3 pollution is typically more pronounced. Effect estimates were also expressed per interquartile range increase in O_3_ to facilitate interpretation on an alternative exposure scale. For the temperature–response association, we similarly increased the df for the temperature and RH splines from 3 to 4 and excluded PM_2.5_ from the model.

## 3 Results

### 3.1 Characteristics of outpatient visits and environmental exposures

A total of 43,418 outpatient visits for allergic rhinitis during April–October of 2013–2023 were included. These visits were matched to 147,453 control-day observations, yielding 43,418 case-crossover strata and 190,871 case- and control-day observations. Of the included visits, 23,477 occurred among males (54.1%) and 19,935 among females (45.9%), with sex missing for 6 visits. Most visits occurred among patients aged 18–64 years (33,592, 77.4%), followed by those aged <18 years (7,999, 18.4%) and ≥65 years (1,822, 4.2%); age was missing for 5 visits.

Environmental exposure distributions on case and control days are summarized in Table 1. Mean O_3_ concentrations were slightly higher on case days (127.3 ± 38.7 μg/m^3^) than on control days (124.7 ± 38.4 μg/m^3^), whereas mean PM_2.5_ concentrations were similar (41.4 and 41.9 μg/m^3^). Case days also had slightly higher temperatures and lower RH than control days. Across all case and control days, the lag 0–1 mean temperature ranged from 2.1 °C to 35.2 °C, indicating that the April–October study period covered a broad range of warm-season temperature conditions in Henan.

**Table 1:** Distribution of environmental exposures on case and control days.

Variable	Day type	Mean ±SD	Min	*P*_25_	*P*_50_	*P*_75_	Max
O_3_ (μg/m^3^)	Control day	124.7 ± 38.4	12.5	97.9	125.4	151.6	247.1
	Case day	127.3 ± 38.7	13.6	101.2	128.5	153.4	247.1
Tm (°C)	Control day	22.5 ± 5.2	3.7	18.9	23.2	26.4	35.2
	Case day	22.7 ± 5.1	2.1	19.3	23.5	26.3	35.0
PM_2.5_ (μg/m^3^)	Control day	41.9 ± 23.1	1.0	26.0	36.5	52.0	200.5
	Case day	41.4 ± 23.0	1.0	25.5	36.4	51.0	200.5
RH (%)	Control day	62.3 ± 15.5	13.6	50.8	63.3	74.3	98.2
	Case day	60.6 ± 15.6	14.8	49.0	61.5	72.8	97.0

Spearman correlation coefficients among the main environmental variables are presented in Supplementary Figures S2, S3. O3 was moderately positively correlated with temperature (Spearman's ρ = 0.54, Supplementary Figure S2), inversely correlated with RH (ρ = −0.31), and weakly correlated with PM_2.5_ (ρ = −0.08).

### 3.2 Association between O3 exposure and outpatient visits for allergic rhinitis

After adjustment for temperature, RH, and PM_2.5_, each 10 μg/m^3^ increase in O_3_ was associated with a 1.2% increase in the odds of an outpatient visit for allergic rhinitis in the overall population (OR = 1.012, 95% CI: 1.006–1.017; Table 2). When expressed per interquartile range increase in O3 (53.4 μg/m^3^), the corresponding OR was 1.064 (95% CI: 1.032–1.097; Supplementary Table S1). Additional lag analyses showed generally positive associations across single-day lags, with a relatively higher OR around lag 6, followed by a gradual decline at longer lags (Supplementary Figure S4A). In the moving-average analyses, the ORs generally increased with longer averaging windows and tended to stabilize at longer lag windows (Supplementary Figure S4B). The non-linear exposure–response analysis showed that the lowest predicted odds occurred at an O3 concentration of approximately 110 μg/m^3^. Using 110 μg/m^3^ as the reference concentration, the estimated ORs were 1.047 (95% CI: 0.977–1.122), 1.013 (95% CI: 0.986–1.040), 1.032 (95% CI: 0.999–1.067), and 1.181 (95% CI: 1.115–1.250) at 50, 80, 150, and 200 μg/m^3^, respectively, indicating a more pronounced increase in the association at higher O3 concentrations, particularly above approximately 150 μg/m^3^ (Supplementary Figure S5).

**Table 2:** Overall and subgroup-specific associations between O_3_ exposure and allergic rhinitis-related outpatient visits.

Group	No. of case/control-day observations	No. of cases	OR (95% CI)
Overall	190,871	43,418	1.012 (1.006–1.017)
Sex			
Female	87,580	19,935	1.011 (1.003–1.020)
Male	103,264	23,477	1.012 (1.004–1.020)
Age			
<18	35,295	7,999	1.030 (1.017–1.044)
18–64	147,539	33,592	1.008 (1.002–1.015)
≥65	8,014	1,822	0.984 (0.957–1.011)

Observation counts include both case and matched control days; missing sex or age in case visits corresponded to 27 and 23 case/control-day observations, respectively.

Positive associations were observed in both females (OR = 1.011, 95% CI: 1.003–1.020) and males (OR = 1.012, 95% CI: 1.004–1.020). The O_3_-related estimate was larger among patients aged <18 years (OR = 1.030, 95% CI: 1.017–1.044). A weaker positive association was observed among those aged 18–64 years (OR = 1.008, 95% CI: 1.002–1.015), whereas no clear association was observed among patients aged ≥65 years (OR = 0.984, 95% CI: 0.957–1.011). Formal interaction tests showed evidence of heterogeneity across age groups (χ^2^ = 25.15, df = 2, *P* < 0.001).

### 3.3 Temperature–response association with outpatient visits for allergic rhinitis

A non-linear temperature–response pattern was observed in the overall population (Figure 2). The MRT estimated from the full-sample model was 15.5 °C. Relative to the MRT, the odds of an outpatient visit for allergic rhinitis increased with rising temperature, peaked at approximately 24.3 °C (OR = 1.336, 95% CI: 1.274–1.401), and subsequently declined at higher temperatures.

**Figure 2:**
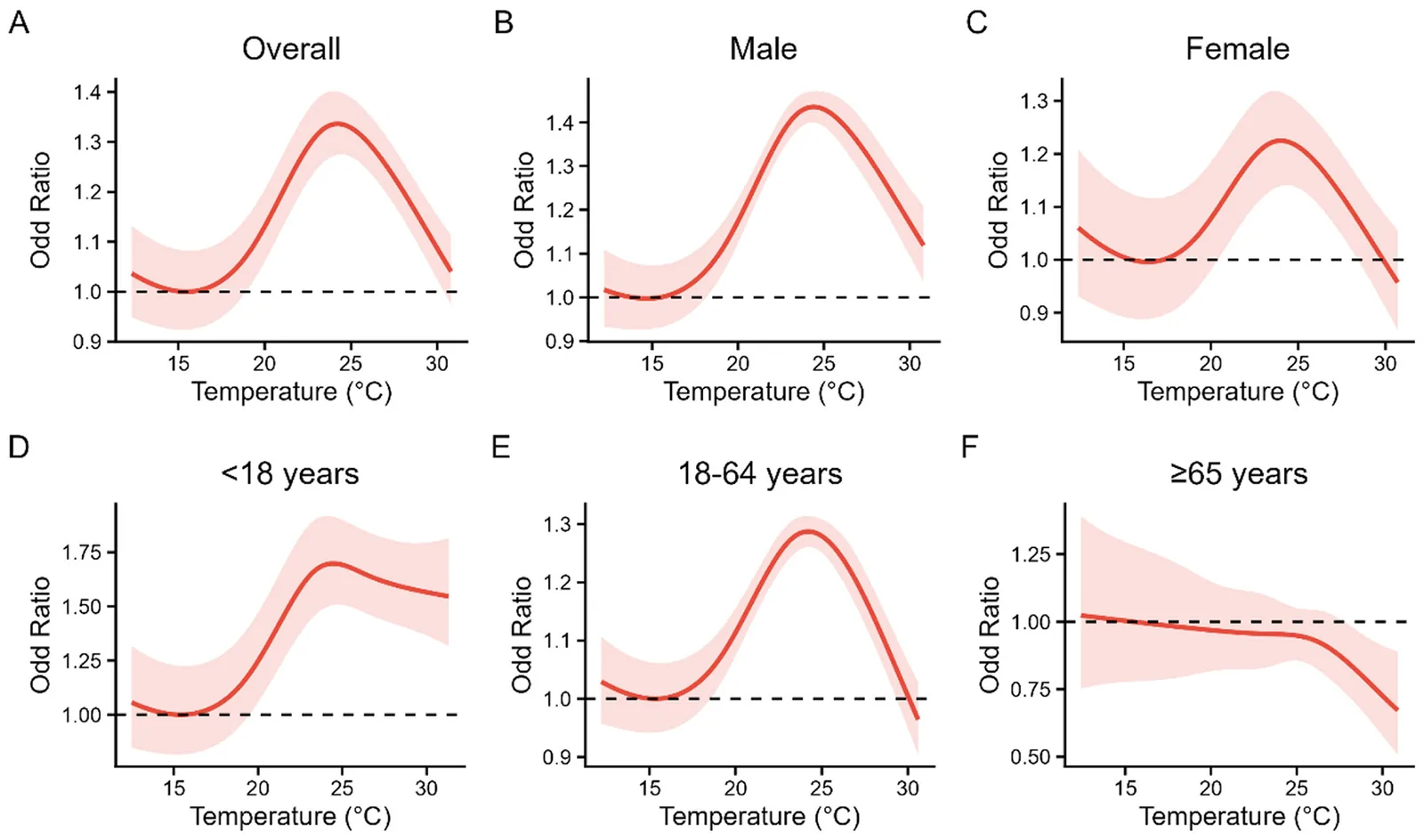
Temperature–response association with allergic rhinitis-related outpatient visits.

Similar inverted U-shaped patterns were observed in males and females. Among males, the OR peaked at approximately 24.4 °C (OR = 1.435, 95% CI: 1.399–1.471), whereas the peak OR was 1.225 (95% CI: 1.140–1.316) in females.

Age-specific curves showed different patterns. Among patients aged <18 years, the OR increased markedly with temperature, peaked at approximately 24.5 °C (OR = 1.698, 95% CI: 1.510–1.914), and remained elevated at higher temperatures. The pattern among patients aged 18–64 years was similar to that in the overall population, with higher odds mainly at moderately high temperatures. Among patients aged ≥65 years, no clear increase was observed, and the wide confidence intervals indicated substantial uncertainty.

### 3.4 Effect modification of the O_3_ association by temperature

The model including the interaction between O3 and the 3-df temperature spline provided a significantly better fit than the model without the interaction terms (likelihood-ratio χ^2^ = 31.2, df = 3, *P* < 0.001), indicating that the O3 association varied significantly across temperature levels.

In the overall population, the O_3_-related OR generally increased with rising temperature (Figure 3). The OR per 10 μg/m^3^ increase in O_3_ was associated with an OR of 1.000 (95% CI: 0.990–1.010) at the 25th percentile of temperature, 1.014 (95% CI: 1.005–1.022) at the 50th percentile, and 1.021 (95% CI: 1.013–1.029) at the 75th percentile (Table 3). Similar increasing patterns were observed in males and females, with O_3_-related ORs at the 75th percentile of temperature of 1.020 (95% CI: 1.009–1.030) and 1.022 (95% CI: 1.011–1.034), respectively.

**Figure 3:**
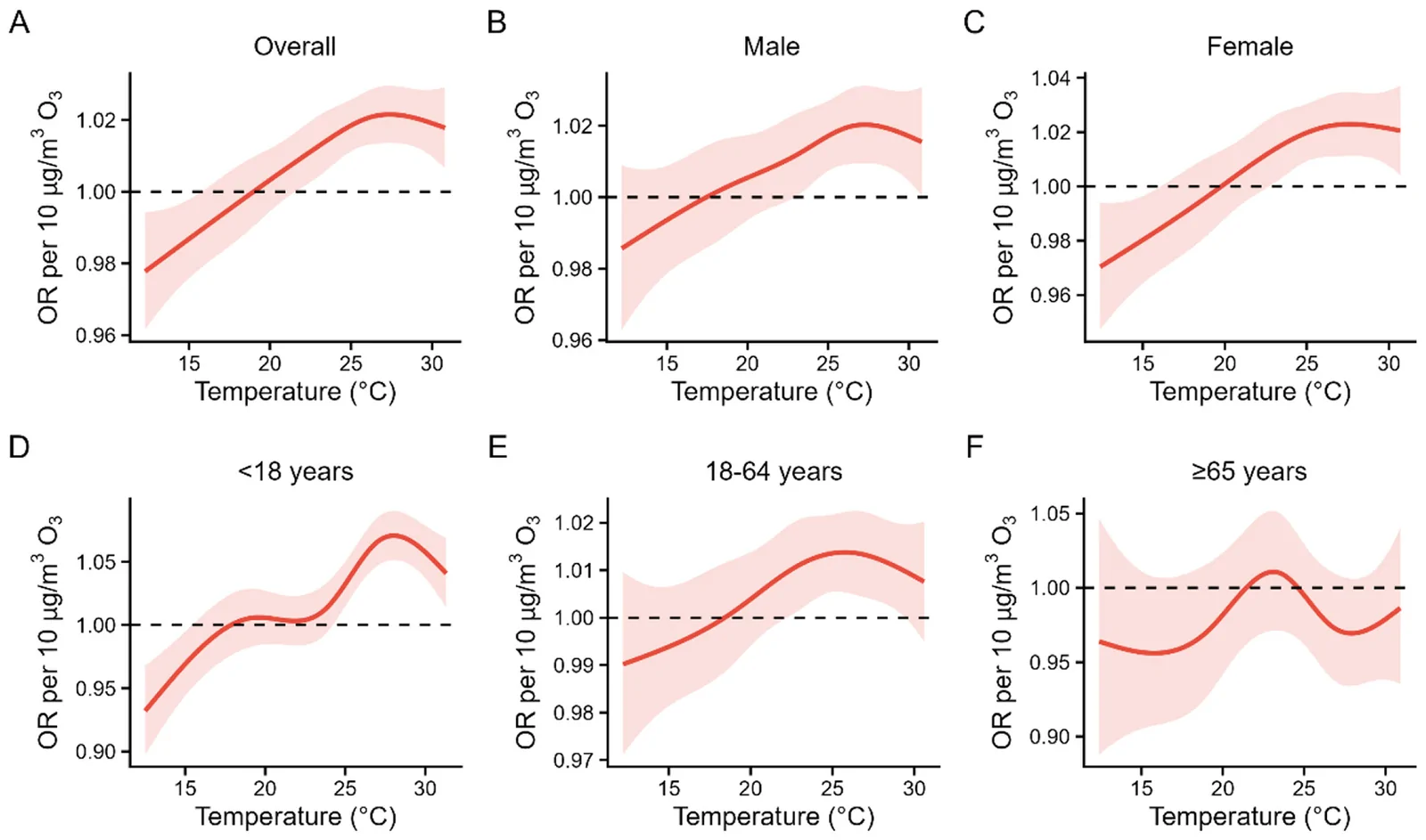
Temperature-dependent associations between O_3_ exposure and outpatient visits for allergic rhinitis.

**Table 3:** Associations between O_3_ exposure and allergic rhinitis-related outpatient visits at selected temperature percentiles.

Group	Tm percentile	Tm (°C)	OR (95%CI)
Overall	25%	19.0	1.000 (0.990, 1.010)
	50%	23.3	1.014 (1.005, 1.022)
	75%	26.4	1.021 (1.013, 1.029)
Sex			
Female	25%	19.1	0.997 (0.983, 1.011)
	50%	23.3	1.015 (1.003, 1.027)
	75%	26.4	1.022 (1.011, 1.034)
Male	25%	18.9	1.003 (0.990, 1.017)
	50%	23.2	1.012 (1.001, 1.024)
	75%	26.4	1.020 (1.009, 1.030)
Age			
<18	25%	19.2	1.006 (0.983, 1.029)
	50%	23.8	1.013 (0.994, 1.033)
	75%	27.0	1.065 (1.046, 1.084)
18–64	25%	18.9	1.001 (0.990, 1.012)
	50%	23.1	1.011 (1.002, 1.021)
	75%	26.2	1.014 (1.005, 1.022)
≥65	25%	19.1	0.971 (0.928, 1.016)
	50%	23.5	1.010 (0.971, 1.050)
	75%	26.7	0.974 (0.939, 1.010)

Age-specific analyses showed that the temperature-dependent pattern was more pronounced among patients aged <18 years. In this group, the O3-related OR increased from 1.006 (95% CI: 0.983–1.029) at the 25th percentile of temperature to 1.065 (95% CI: 1.046–1.084) at the 75th percentile (Figure 3). A weaker increase was observed among patients aged 18–64 years, for whom the corresponding ORs were 1.001 (95% CI: 0.990–1.012) and 1.014 (95% CI: 1.005–1.022). Among patients aged ≥65 years, no consistent positive pattern was observed (Figure 3). The three-way interaction among O3, the temperature spline, and age group was statistically significant (χ^2^ = 34.55, df = 6, *P* < 0.001), indicating that temperature modification of the O_3_ association differed across age groups.

### 3.5 Joint exposure–response association between temperature and O_3_

Figure 4 shows the model-predicted associations between temperature and outpatient visits for allergic rhinitis at different fixed O_3_ levels. In the overall population, the temperature–response curves shifted upward as O_3_ increased, indicating higher odds of outpatient visits under elevated O_3_ conditions, particularly at moderately high temperatures. At 25 °C, the OR increased from 1.147 (95% CI: 1.108–1.187) when O_3_ was fixed at its 25th percentile to 1.241 (95% CI: 1.170–1.317) when O_3_ was fixed at its 75th percentile.

**Figure 4:**
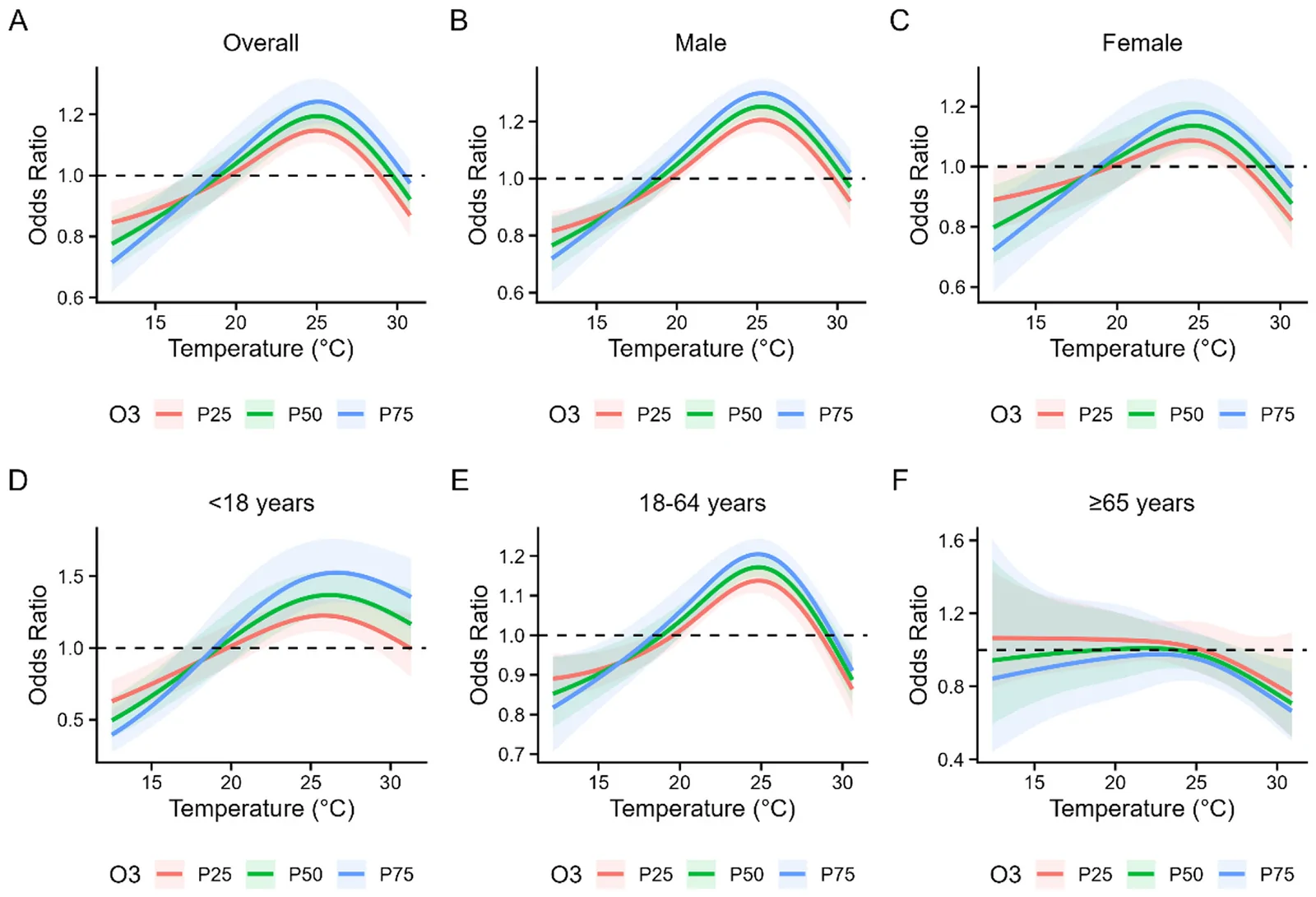
Model-predicted associations between temperature and outpatient visits for allergic rhinitis at different O_3_ levels.

Similar patterns were observed in males and females, with higher temperature-related ORs at higher O_3_ levels. Age-stratified analyses showed that this pattern was most evident among patients aged <18 years. In this group, the OR at 25 °C increased from 1.220 (95% CI: 1.114–1.336) at the 25th percentile of O_3_ to 1.499 (95% CI: 1.289–1.744) at the 75th percentile. A similar but weaker pattern was observed among patients aged 18–64 years.

### 3.6 Sensitivity analyses

Sensitivity analyses supported robustness of the main findings (Supplementary Table S1). The ORs per 10 μg/m^3^ increase in O_3_ were 1.013 (95% CI: 1.007–1.018) when 4 df were used for the temperature spline, 1.012 (95% CI: 1.006–1.017) when 4 df were used for RH, and 1.016 (95% CI: 1.009–1.023) when the analysis was restricted to May–September. An interquartile range increase in O_3_ (53.4 μg/m^3^) was associated with an OR of 1.064 (95% CI: 1.032–1.097). The temperature–response curves were also largely unchanged under alternative spline specifications and after excluding PM_2.5_ adjustment, consistently showing an inverted U-shaped pattern (Supplementary Figure S6).

## 4 Discussion

The results of this study suggest that short-term ambient O_3_ exposure was associated with higher odds of rhinitis-related outpatient visits, with stronger associations observed at higher temperatures. These findings indicate that ambient temperature may act not only as a potential confounder but also as an effect modifier in the O_3_-rhinitis association, highlighting the need to consider temperature when evaluating O_3_-related upper-airway health effects. However, the decline in the temperature–response curve at very high temperatures should not be interpreted as evidence of a protective effect. This pattern may partly reflect behavioral adaptation, reduced outdoor activity, air-conditioning use, changes in healthcare-seeking behavior, or fewer observations at very high temperatures.

Previous studies have linked O_3_ and other air pollutants to allergic rhinitis and related upper-airway outcomes, suggesting that air pollution may exacerbate rhinitis symptoms and increase related healthcare utilization (2, 3). However, findings remain inconsistent, likely due to differences in study populations, exposure windows, outcome definitions, and confounder adjustment. A German birth cohort study also observed a positive association between O_3_ exposure and allergic rhinitis, although the findings varied across study regions (18). Our study extends previous evidence by showing that the short-term association between warm-season O_3_ exposure and rhinitis-related outpatient visits varies by ambient temperature. The non-linear analysis further suggested that the O3–rhinitis association varied across the exposure range, rather than increasing at a constant rate. This finding is consistent with previous studies showing that O3-related respiratory effects may exhibit non-linear concentration–response relationships (19). The stronger association observed at higher O3 concentrations may partly reflect increased oxidative stress and airway epithelial injury with increasing O3 exposure (20).

Biologically, the association between O_3_ exposure and rhinitis-related outpatient visits is plausible. As a highly reactive oxidant gas, inhaled O_3_ can react with airway lining fluid and epithelial cell membranes, generating reactive oxygen species and lipid oxidation products that may trigger oxidative stress, epithelial barrier dysfunction, increased mucosal permeability, and local inflammation (7). Because the nasal cavity is one of the first contact sites for inhaled O_3_, the nasal mucosa may be particularly vulnerable to O_3_-induced oxidative injury. Recent studies have identified epithelial barrier dysfunction as a key pathway by which pollutants, allergens, and pathogens may promote allergic inflammation (21, 22). Animal studies have further shown that O_3_ inhalation may exacerbate eosinophilic inflammation and Th2-skewed immune responses in allergic rhinitis models, while also affecting Nrf2-related antioxidant responses and oxidative stress in the nasal mucosa (23, 24). In addition, a panel study of patients with allergic rhinitis suggested that short-term exposure to air pollution mixtures was associated with increased nasal oxidative stress and symptom worsening, with O_3_ identified as a potential contributor (25). Together, these findings suggest that oxidative stress, epithelial barrier disruption, and allergic inflammation may jointly contribute to O_3_-related aggravation of rhinitis symptoms.

Ambient temperature significantly modified the association between O_3_ exposure and rhinitis-related outpatient visits, with higher O_3_-related ORs observed at higher temperatures. This pattern may reflect both enhanced O_3_ formation under warmer conditions and increased upper-airway susceptibility. Higher temperature and stronger solar radiation can promote photochemical O_3_ formation, increasing the likelihood of concurrent heat and O_3_ exposure (5). Heat may also increase ventilation, promote airway surface dehydration, and aggravate nasal mucosal irritation, potentially making the upper airway more susceptible to O_3_ exposure. Previous epidemiological and experimental studies support potential joint effects of temperature and O_3_ on respiratory or allergic outcomes (9, 10, 26). These findings suggest that ambient temperature should be considered as both a potential confounder and an effect modifier when evaluating O_3_-related upper-airway health effects.

Age-stratified analyses showed larger O_3_-related estimates and a more apparent temperature-dependent pattern among patients aged <18 years. This finding may reflect differences in biological susceptibility, exposure behavior, and healthcare-seeking patterns. Children and adolescents have developing respiratory and immune systems, higher ventilation rates relative to body weight, and potentially more outdoor activity during the warm season, which may lead to a higher relative inhaled dose under the same ambient exposure level (11). Parents may also be more likely to seek medical care when children develop acute nasal symptoms, which may partly contribute to the stronger associations observed in outpatient data. Previous studies also support children and adolescents as potentially susceptible populations for O_3_-related allergic or respiratory health effects (12, 13). In contrast, no consistent positive pattern was observed among patients aged ≥65 years. This finding should be interpreted cautiously because the number of cases in this subgroup was relatively small, which may have limited statistical power. Moreover, rhinitis symptoms in older adults may be more heterogeneous, with a greater contribution of non-allergic rhinitis, comorbidities, medication use, and differences in healthcare-seeking behavior, which may have attenuated or obscured the O_3_-related association. Therefore, the absence of a consistent positive association in this subgroup should not be interpreted as evidence of no O_3_-related effect in older adults.

Temperature, RH, and PM_2.5_ were included as covariates because they may be related to both O_3_ concentrations and rhinitis-related outpatient visits. Temperature can influence photochemical O_3_ formation and may also directly affect nasal mucosal responses (5). RH may affect airway irritation, allergen behavior, pollutant dispersion, and human comfort; previous studies have also suggested that temperature and RH may modify the association between air pollution and allergic rhinitis outpatient visits (3). PM_2.5_ may independently induce respiratory inflammation and allergic responses and has been associated with rhinitis symptoms and other allergic diseases (2, 4). Therefore, adjustment for temperature, RH, and PM_2.5_ in the main model helped reduce potential confounding by meteorological conditions and co-pollutant exposure when estimating the O_3_ association. However, this study focused on the short-term association of O_3_ exposure and its modification by temperature, and did not separately evaluate the independent health effects of particulate matter.

These findings have potential public health implications. As warm-season O_3_ pollution becomes increasingly prominent in China (6), health risk assessment based only on pollutant concentrations may not fully capture high-risk conditions. Our results suggest that O_3_-related rhinitis outpatient visits may be more likely under higher temperature conditions, especially among children and adolescents. Therefore, warm-season O_3_ health warnings and protection strategies should incorporate ambient temperature and pay particular attention to susceptible groups.

Several limitations should be noted. First, exposure misclassification may have occurred because environmental exposures were assigned according to residential address, without information on personal mobility or indoor exposure. Second, data on pollen, bioaerosols, viral infections, indoor allergens, and other environmental irritants were unavailable. Pollen is a major trigger of allergic rhinitis and may vary with temperature and season. Therefore, residual confounding by pollen may have partly influenced the observed temperature-dependent O_3_ association. Third, although PM_2.5_ was adjusted for in the main model, we did not separately assess the independent effects of particulate matter, and PM_10_ was not included because it was highly correlated with PM_2.5_. In addition, NO_2_, SO_2_, and CO are gaseous co-pollutants and were not included in the main model. Finally, the outpatient data were obtained from a single large hospital, which may limit the generalizability of the findings.

## 5 Conclusions

Short-term exposure to ambient O_3_ was associated with higher odds of allergic rhinitis-related outpatient visits during the warm season in Henan Province, China. Each 10 μg/m^3^ increase in lag 0–1 O_3_ was associated with a modest increase in outpatient visits, and the association became more apparent over a larger exposure contrast. The O_3_ association was stronger at higher temperature levels and showed a more apparent temperature-dependent pattern among children and adolescents, suggesting that this population may represent a potentially susceptible group during warm-season O_3_ episodes. These findings highlight the importance of considering ambient temperature when evaluating O_3_-related upper-airway health effects and support integrated public health strategies for reducing combined exposure to O_3_ and heat.

## Data Availability

The original contributions presented in the study are included in the article/Supplementary material, further inquiries can be directed to the corresponding author.
