# Supplementary material for: Temperature-dependent association between ambient ozone exposure and allergic rhinitis-related outpatient visits: a time-stratified case-crossover study in Henan Province, China
Source: Front Public Health. 2026 Oct 20;14:1952329. doi: 10.3389/fpubh.2026.1952329 (PMC13727516; doi:10.3389/fpubh.2026.1952329)
Supplement: Supplementary file 1 [file Data_Sheet_1.docx]

Temperature-Dependent Association between Ambient Ozone Exposure and Allergic Rhinitis-Related Outpatient Visits: A Time-Stratified Case-Crossover Study in Henan Province, China

Dong Ding^1#^, Wei Lu^2 #^, Huina Wang^3^, Leiyu Wang^4^, Jinghong Gao^1,2^, Tengda Wei^2*^, Haohao Liu^2^^*^

^1^ College of Public Health, Zhengzhou University, Zhengzhou, 450001, Henan, China

^2^ Department of Public Health, The First Affiliated Hospital of Zhengzhou University, Zhengzhou, 450001, Henan, China

^3^ School of Public Health, Cheeloo College of Medicine, Shandong University, Jinan, Shandong, China.

^4^ Boston University, Commonwealth Ave, Boston, MA 02215

\# These authors have contributed equally to this work and share first authorship

* Corresponding authors:

Haohao Liu

**E-mail**: liuhaohao@zzu.edu.cn

Tengda Wei

**E-mail**: td_wei@163.com

Fig. S1. Flowchart of outpatient visit selection and construction of the time-stratified case-crossover dataset.

Fig. S2 Association between temperature and O_3_ concentration

Fig. S3. Spearman correlation matrix among the main environmental variables.

Fig. S4. Associations between O₃ exposure and allergic rhinitis-related outpatient visits across single-day lags and moving-average lag windows.

Fig. S5. Nonlinear exposure–response association between O₃ and allergic rhinitis-related outpatient visits.

Fig. S6. Sensitivity of the Temperature–Rhinitis Exposure–Response Association to Alternative Spline Degrees of Freedom.

Table S1. Associations between O₃ exposure and allergic rhinitis-related outpatient visits per interquartile range increase in O₃.

| **Group** | **Level** | **No. of observations** | **No. of cases** | **OR (95% CI)** |
| --- | --- | --- | --- | --- |
| Overall | Overall | 190,871 | 43,418 | 1.064 (1.032–1.097) |
| Sex | Female | 87,580 | 19,935 | 1.063 (1.016–1.111) |
| Sex | Male | 103,264 | 23,477 | 1.066 (1.023–1.111) |
| Age | <18 | 35,295 | 7,999 | 1.171 (1.093–1.255) |
| Age | 18–64 | 147,539 | 33,592 | 1.044 (1.008–1.081) |
| Age | ≥65 | 8,014 | 1,822 | 0.916 (0.791–1.061) |
